# Nurse students’ experiences with clinical placement in outpatient unit – a qualitative study

**DOI:** 10.1186/s12912-016-0167-1

**Published:** 2016-08-08

**Authors:** Ann Karin Helgesen, Anne Grethe Gregersen, Anne Karine Østbye Roos

**Affiliations:** 1 Faculty of Health and Social studies, Østfold University College, P.O. Box 700, NO 1757 Halden, Norway; 2 Nursing Department, Østfold Trust Hospital, P.O. Box 300, 1714 Grålum, Norway

**Keywords:** Nursing education, Hospital, Outpatient unit, Reflection, Learning outcomes, Conventional analyses

## Abstract

**Background:**

The recent reforms in the health care sector have changed the requirements for professional nursing competence in the clinical field. The reforms have also required nursing education to consider different areas for clinical placements for their students, and outpatient units in hospitals have been increasingly formalized as clinical learning environments. The complex technologies in some of these units represent a challenge for students who have limited existing knowledge or experience. More focus on outpatient care has also led to fewer opportunities for studying the continuity of a patient’s life situation. In order to meet these challenges, structured learning activities with special forms were developed by nursing educators and nurses at outpatient units. The aim of this study was to explore students' experiences of using structured learning activities as unit-specific learning outcomes and targeted reflection during clinical placements in an outpatient unit.

**Methods:**

Two focus group interviews were conducted with a total of seven nursing students who had experienced structured learning activities during clinical placements in an outpatient unit. Data were analyzed by means of content analyses.

**Results:**

This study shows that preparedness and guidance during placement were imperative for making the week in the outpatient unit meaningful. ‘Being prepared’, which was one of the categories, incorporated the subcategories ‘being able to understand what to do’, ‘being at the right place at the right time’ and ‘being alert for new experiences’. The category ‘being guided’ which incorporated the subcategories ‘from uncertainty to more confidence’, ‘from observer to seeking knowledge’ and ‘from focusing on technology to seeing the person’ showed that the forms guided the students through the placement in the outpatient unit.

**Discussion:**

Students take a more active approach to seeking knowledge when given structured learing activities during clinical placement in outpatient unit.

**Conclusions:**

This study shows that use of outpatient units for clinical placement in nursing studies has several challenges but also the potential for creating positive experiences for the students.

## Background

Over the past decade, there has been a reduction of hospital beds in most European countries. Across EU member states, the ‘Health at a glance – Europe 2012’ report asserts a decrease in hospital beds from 6.5 beds per 1000 population in 2000 to 5.3 in 2010. The development of new and improved technology has enabled a transition in most countries from long hospitalizations to same-day surgeries and increased use of outpatient treatments [1]. In Norway there has been a 6 % reduction in hospital beds from 2010 to 2014 [2]. The introduction of the Coordination Reform [3] in 2012 saw a restructuring of acute care and an increased focus on outpatient treatment. This has led to a sustained investment in outpatient treatment facilities and ambulatory care [2].

Fifty percent of nursing education in Norway consists of clinical practice where the students are under the supervision of registered nurses. The intention of clinical practice is to acquire basic nursing skills in a natural environment [4]. The number of nursing students rose by 14 % between 2010 to 2014, from 3,179 to 3,630 [5].

The decrease in the number of hospital beds in parallel with the increase in the number of nursing students has created a challenge in terms of providing the best possible learning facilities under given conditions in hospitals.

Along with substantial economic restrictions around the world [6], the recent reforms in the health sector [3] have changed requirements for professional nursing competence in the clinical field. Rapid progress in the treatment of many medical conditions has caused a shift in nurses’ duties and functions and many changes in nursing education over the last two decades [7, 8]. The nursing education sector has had to rethink the knowledge and skills it provides and how it teaches patient interaction to its students [9]. The reforms have also required nursing education to consider different areas for clinical placements for its students, and outpatient units in hospital have been increasingly formalized as clinical learning environments [10]. Complex technologies in some of these units represent a challenge for students who have limited existing knowledge or experience. More focus on outpatient care has also led to fewer opportunities for studying the continuity of a patient’s life situation. Although there have been many changes in the health care sector and in the education of nurses, caring is still the essence of nursing and has been described as an ‘*intricate interplay in the care context’* [11]. The art of caring should therefore be emphasized both in theory and in clinical practice. To maintain this association, it is important that the clinical placement offers an environment where this can be taught. Löfmark et al. [7] claim that good co-operation between clinical staff and nurse educators is necessary for providing a good learning environment for nursing students. The importance of preparing students for their clinical practice, including an ability to integrate theory into a clinical setting and to secure time for reflection, to promote learning and confidence, has been highlighted in several studies [12–14]. In order to learn, students need to feel a sense of belonging [15]. The literature suggests that developing a professional identity and an aspiration to learn are influenced by the length of the clinical practice [16]. Short-term placement has been shown to offer students less opportunity to participate in the social interactions that go into making them future professionals [15]. A previous collaboration project between Østfold University College (HiØ) and Østfold Trust Hospital (SØ) in Norway aimed to try out a rotation programme between traditional wards and outpatient units. The evaluation of the programme showed that some outpatient units were unsuitable for providing sufficient relevant learning situations for nursing students [17]. Given these conditions, HIØ teamed up with SØ in 2014 for a new project. Influenced by an ongoing project at Bethesda North called the ‘Off ward shadowing experience’ [18] a project team consisting of nurse educators from HIØ and nurses working at SØ outpatient units developed forms with unit-specific learning outcomes and targeted reflection for eight technologically advanced procedures in cardiology and neurology outpatient units. The forms were designed with the aim of enhancing reflection, consciousness and recognition of basic nursing skills in order to yield a better learning experience from placements in outpatient units. The students spent one week at the outpatient unit out of a total of eight weeks in clinical placement.

## Aim

The aim of this study was to explore students' experiences of structured learning activities using forms for unit-specific learning outcomes and targeted reflection during clinical placements in outpatient units.

## Methods

The study has a qualitative explorative design. Conventional content analysis following Hsieh and Shannon [19], also described as inductive category development [20], was used.

### Settings and accession to the field

The study was conducted in a hospital in the eastern part of Norway. Written permission was obtained from the head of the hospital nursing department and the head of the outpatient department before the study was conducted. The second-year students undergoing clinical placement were informed by two instructors in the upcoming program that a study would take place in the outpatient units and that some of them would be contacted and asked if they wanted to participate in the study. Students could decline to be further contacted.

### Informants

Inclusion criteria for joining the study were: second-year students, undergoing clinical placement at the cardiology or neurology units. Nine students were asked to participate in the study, but only seven ultimately participated in the interviews. The time scheduled for the interviews was changed shortly before they were due to take place and two students failed to notice the change. The age of the informants, six women and one man, ranged between 20 and 41 years (mean 28, median 22). Four of them undertook clinical placement at the cardiology unit and three of them at the neurology unit.

### Data collection

Data were collected by means of focus group interviews during spring 2015. The first group consisted of five informants. The second group consisted of only two informants, as two informants did not show up for interview. An interview guide, with themes concerning the informants’ experiences with the forms’ unit-specific learning outcomes and targeted reflection in the outpatient unit, was used. The questions were inspired by the content of these forms. The interviews started by obtaining biographical data and with open dialogue during which the informants were encouraged to talk freely about their experiences (e.g. asking about their experiences of the use of unit-specific learning outcomes). The interview guide was used as a reminder to the researchers to ensure that the themes were covered. In order to obtain rich and meaningful data, probing questions were sometimes asked by both researchers to extend or narrow the field of interest (e.g. ‘could you please elaborate on this?’).

The interviews took place in a quiet room at the hospital, and, with the exception of a phone call, they were conducted without interruption. They took place at the end of the informants’ clinical placements. As author^2^ was also a nurse educator for the informants, author^1^, who was unknown to the informants, was in charge of the interviews. During the final 30 min approximately, author^2^ left the room so that her presence did not hinder the informants from talking freely about their experiences. At the very end of the interviews, which were each 50 min long, a verbal summary of the content was presented to the informants to ensure that their experiences had been correctly understood.

### Analysis

Immediately after the interviews, notes were made by author^1^ to capture the setting and reflections in respect of the informants’ emotional responses. The interviews without any biographical details of the informants were transcribed verbatim by a secretary and delivered to the three authors. The authors read the interviews right through each by themselves in order to obtain a sense of the whole. Thereafter the interviews were read word by word. Words which were interpreted to capture key experiences of the informants were highlighted in the text and assigned preliminary codes [19]. In the next step of the process, the research team met and compared their codes and sorted them into categories and subcategories. Quotations were selected to support each description and to secure the trustworthiness of the data.

## Results

Two categories and 6 subcategories, describing students’ experiences of using forms for unit-specific learning outcomes and targeted reflection in outpatient units, were identified (Table 1).

**Table 1:** Students’ experiences of using forms for unit-specific learning outcomes and targeted reflection in outpatient units

Categories	Subcategories
Being prepared	Being able to understand what to do
	Being at the right place at the right time
	Being alert to new experiences
Being guided	From uncertainty to more confidence
	From observer to seeking knowledge
	From focusing on technology to seeing the person

### Being prepared

Data revealed that the students’ preparedness when entering the outpatient unit impacted on how they described the week. A high level of preparedness seemed to be close to a prerequisite in order for a student to perceive the week as successful.

#### Being able to understand what to do

Data revealed that some of the students lacked comprehensive understanding about being part of this specific project, of the terms used and about the forms. This led to frustration and distress.

*‘It has something to do with information failure. When I wasn’t informed like I was supposed to about how to use the forms, I guess I wasn’t able to benefit from them as intended.’*

#### Being at the right place at the right time

Data also revealed that the timing of the students’ placements in the outpatient unit impacted on their preparedness, as they had to familiarize themselves with the group of patients and certain concepts before entering the unit.

*‘My week at the outpatient unit, was in my second week of clinicals. I had been working two shifts at the ward, so there was a lot going on in my head already, so I was maybe a little stressed because I knew absolutely nothing when I came there, so I thought that was a little senseless. I wish I could have spent more time at the ward, so at least I knew some of the terminology.’*

Students who had their week at the outpatient unit at the end of their clinical placement period perceived that they achieved better learning outcomes from their placements than the other students.

#### Being alert to new experiences

Data showed that some of the students had read and used the forms ahead of the week in the outpatient unit. When having used the given tools, data indicated that students felt a higher ability to focus on their upcoming practice.

*‘The forms were useful in terms of preparing for the procedures and to define what we were supposed to be focusing on at the outpatient clinic.’*

As this was their first encounter with an outpatient clinic and such procedures, using the forms and preparing ahead enabled students to be more alert to new experiences. Students who did not use the forms, expressed less alertness.

### Being guided

Data showed that the forms guided the students through their placements in the outpatient unit.

#### From uncertainty to more confidence

There are waiting periods between most procedures at outpatient units. The students reported uncertainty concerning proper use of time. The forms seemed to give the students more confidence as they had something meaningful to do between procedures.

*‘If I did not have those forms to help me reflect, I would never have understood what I was actually doing or was a part of.’*

*‘The fact that you are being guided towards what you are supposed to look for and stuff, I really liked it.’*

#### From observer to seeking knowledge

Compared to the regular ward which was perceived as more active, the outpatient placement was described as a relatively passive learning environment. Instead of just observing, the forms helped the students take a more vigorous approach in seeking knowledge.

*‘It was often like you could just sit down in a nook and answer the questions because it wasn’t really busy.’*

#### From focusing on technology to seeing the person

Data revealed the challenges of being a student in a highly technological world. The forms directed the students’ attention towards the patient and their experiences and the relationships between the nurses and the patient.

*‘The questions in the form made me look away from all the equipment they have at the outpatient unit, they made me more focused on the patients and made me think more about how the patients were being informed.’*

## Discussion

This study shows that preparedness and guidance during placement were essential to making the week in the outpatient unit meaningful. Some of the findings agree with other studies that have found that using outpatient units as clinical placements in nursing studies has several challenges [17]. However the findings also revealed opportunities for making the placement a positive experience for the students.

An interesting finding was that, although both written and oral information was given by the nurse educators, some of the students were almost unaware of being part of a project, of the concepts being used and how to use and to fill out the forms. Other students were well prepared for the new experiences, as they had read and used the forms ahead of the week in the outpatient unit in order to be alert. Taking responsibility for one’s own learning is an important part of being prepared [13] and being prepared for clinical placement is an attribute that can separate the successful students from the unsuccessful ones. Previous literature indicates however that students may not actually comprehend how to effectively prepare for their clinical placement [12, 13], which might also be the issue among some of the students in this study. Some students highlighted information failure and a lack of communication of expectations as reasons for not understanding what to do. This information failure can most likely be linked to the introduction of the new concepts of learning outcome and targeted reflection, and the new forms which had been specifically developed for the project. The concepts and forms were well-known by the nurse educators and preceptors who developed them, but brand new to the students and may have caused frustration and distress. This shows that new concepts and the use of new forms must be explained in detail, since an understanding of what to do is an important part of being prepared. This is consistent with findings in Lewallen and DeBrew’s studies [13, 21].

Another important part of being prepared was linked to being in the right place at the right time. This study shows that it could be hard for students to leave the ward with which they have barely had time to familiarize themselves, and readjust to a new field. Given that students often feel lost and uncertain in the initial phase of clinical placement, time plays an important factor in their experience of a good learning process [16]. It is noteworthy that students did not see it as a problem to undertake a rotation at the end of their clinical placement. However a rotation between an outpatient unit and a regular ward might be challenging, as a key influence on nursing students’ sense of belonging relates to the length and organization of the clinical placement [16]. It is a matter of fact that nursing students gradually advance from understanding the general aspects to the more complex areas of nursing [12, 22]. Newton and McKenna [23] refer to this as a “transitional journey.” Our findings might indicate that, if the students do not feel well adjusted to the general clinical area, their transition to the advanced outpatient unit is more difficult. Newton and McKenna [23] argue that students are often not ready to understand the expertise and advanced treatments that take place in such specialist areas.

In an advanced clinical setting with complex technology and treatments, it can be difficult for inexperienced nursing students to actively seek knowledge. Students may not feel adequately prepared for the multiple challenges, and it can be difficult to know how to approach this.

Chambers, Thiekötter and Chambers [24] claim that nursing education needs to adopt pedagogical approaches that stimulate the learning process, so that students have the necessary ballast to meet the continuous changes taking place in health care. Our findings indicate that, when given a form with questions to answer and settings to reflect upon, students take a more active approach to seeking knowledge. Some students described how the effect of a structured learning process enhanced their placement at the outpatient unit. This may agree with what Coyne and Needham [12] refer to as ‘customizing learning needs.’ Findings also revealed that the forms guided the students’ attention from the technological devices towards the patients and their experiences, and also to the relationship between nurse and patient. This is in line with findings in Adamson and Dewar’s study [25] which emphasizes that reflective learning and the use of stories about the experience of giving and receiving care can contribute to development of knowledge, skill and confidence in the students. Previous research also suggests that learning is stimulated when students conceptualize their experiences and are led into reflecting upon specific situations [26].

Even if clinical placements in outpatient units face several challenges, they also have the potential to offer students positive experiences. Outpatient units traditionally have a higher nurse-patient ratio. Coyne and Needham [12] show that a higher nurse-patient ratio allows more time for student supervision. An outpatient unit is also quite specific in terms of offering students the possibility of observing and participating in a range of different skills. Depending on the outpatient units, students can acquire skills that are essential to nursing practice as well as more specialized knowledge about different examinations and treatments, and an enhanced understanding of the human anatomy, as well as a wider understanding of the nurse’s role. Our findings show that, when in an outpatient clinic, students have more time between patients. This allows time for reflection. Students stated that they found the forms helpful, since they made them use the extra time wisely in reflecting on the procedures they had been observing. Our findings show that, through reflection, students were able to focus less on technology and more on the patient. According to Anderson, Willman, Sjøstrøm-Sand and Berglin [11], the changes occurring in health care place nurses under stress and basic care is not secured. It is therefore vital that students learn to be truly present in order to care for their patients. In our study, the existence of a form to guide the students as to what aspects of nursing are important seems to have had a positive impact on their ability to acknowledge the patient.

### Methodological reflections

Efforts were made to establish an audit trail throughout the entire study. In order to secure as open-minded an approach as possible, the researchers discussed their own preconceptions on the topic and made efforts to put these aside during the data collection and analyses. The researchers were aware that inclusion of more informants might have provided additional data; it was however only these students who underwent this particular experience. In spite of limited numbers of informants in one of the focus group, rich data was provided.

## Conclusions

This study shows that the use of outpatient units for clinical placement in nursing studies faces several challenges. However, the study also identifies opportunities for making the placement in an outpatient unit a positive experience for the students. Preparedness and guidance during placement were imperative for making the week in the outpatient unit meaningful. Students need guidance in understanding concepts and in applying expectations. Customized forms can help students who spend the clinical placement in an outpatient unit see the nursing perspective more clearly. In the future, educators, preceptors and students need to continue developing the concepts and forms. When planning rotation between the regular ward and the outpatient unit, the timing of placement in the outpatient unit should be in the middle or end of the overall clinical placement period. More research is needed on the use of structured learning activities such as unit-specific learning outcomes and targeted reflections in special clinical placements such as outpatient units.

## Abbreviations

HiØ, Østfold University College; SØ, Østfold Trust Hospital
